# Sex Differences in the Pro-Angiogenic Response of Human Endothelial Cells: Focus on PFKFB3 and FAK Activation

**DOI:** 10.3389/fphar.2020.587221

**Published:** 2020-12-17

**Authors:** Carlotta Boscaro, Annalisa Trenti, Chiara Baggio, Chiara Scapin, Lucia Trevisi, Andrea Cignarella, Chiara Bolego

**Affiliations:** ^1^ Department of Pharmaceutical and Pharmacological Sciences, University of Padova, Padova, Italy; ^2^ Department of Medicine, University of Padova, Padova, Italy

**Keywords:** endothelial cells, sex-specific factors, angiogenesis, estrogen, glycolytic enzymes, adhesion molecules, digitoxin

## Abstract

Female hormones and sex-specific factors are established determinants of endothelial function, yet their relative contribution to human endothelium phenotypes has not been defined. Using human umbilical vein endothelial cells (HUVECs) genotyped by donor's sex, we investigated the influence of sex and estrogenic agents on the main steps of the angiogenic process and on key proteins governing HUVEC metabolism and migratory properties. HUVECs from female donors (fHUVECs) showed increased viability (*p* < 0.01) and growth rate (*p* < 0.01) compared with those from males (mHUVECs). Despite higher levels of G-protein coupled estrogen receptor (GPER) in fHUVECs (*p* < 0.001), treatment with 17β-estradiol (E2) and the selective GPER agonist G1 (both 1–100 nM) did not affect HUVEC viability. Migration and tubularization *in vitro* under physiological conditions were higher in fHUVECs than in mHUVECs (*p* < 0.05). E2 treatment (1–100 nM) upregulated the glycolytic activator PFKFB3 with higher potency in fHUVECs than in mHUVECs, despite comparable baseline levels. Moreover, Y576/577 phosphorylation of focal adhesion kinase (FAK) was markedly enhanced in fHUVECs (*p* < 0.001), despite comparable Src activation levels. While the PI3K inhibitor LY294002 (25 µM) inhibited HUVEC migration (*p* < 0.05), Akt phosphorylation levels in fHUVECs and mHUVECs were comparable. Finally, digitoxin treatment, which inhibits Y576/577 FAK phosphorylation, abolished sexual dimorphism in HUVEC migration. These findings unravel complementary modulation of HUVEC functional phenotypes and signaling molecules involved in angiogenesis by hormone microenvironment and sex-specific factors, and highlight the need for sex-oriented pharmacological targeting of endothelial function.

## Introduction

The endothelium is essential in maintaining vascular homeostasis (Cines et al., 1998; Vanhoutte et al., 2017). While the endothelium represents a key target of vascular estrogen action (Bolego et al., 2006a; Bolego et al., 2006b; Pinna et al., 2008; Arnal et al., 2010), endothelial biology is also governed by sex-specific factors (Mudrovcic et al., 2017; Stanhewicz et al., 2018). Moreover, sex hormones exert direct effects and interact with the effects of sex chromosomes during lifetime (Arnold et al., 2017; Ventura-Clapier et al., 2017), suggesting that sex hormones in the local microenvironment as well as intrinsic differences at the cellular level account for gender differences in endothelial function. Thus, understanding the pattern of sex chromosome as distinguished from sex hormone-driven phenotypic changes in the endothelium would be instrumental for the identification of clinically relevant preventive and/or therapeutic strategies.

Due to easy access and high purity, human umbilical vein endothelial cells (HUVECs) are a valuable model for the study of physiopathological processes. Because HUVECs have the fetus’ sex (Annibalini et al., 2014), sex-specific (XX and XY) chromosome-based differences may be explored. For instance, XX and XY cells have different susceptibility to cell death and survival (Malorni et al., 2007). Cultured HUVECs display sexual dimorphism in, e.g., the nitric oxide pathway (Addis et al., 2014; Cattaneo et al., 2017) as well as in the transcriptional profile and intracellular proteome under basal and stress conditions (Lorenz et al., 2015; Lorenz et al., 2019; Witt et al., 2019). Although an association between sex-specific gene variants and coronary endothelial dysfunction has been reported in humans (Yoshino et al., 2016), the relative contribution of sex and estrogenic agents to new vessel formation *in vitro* under physiological conditions remains to be fully characterized.

Endogenous signaling molecules including estrogen can stimulate the repair of damaged blood vessels as well as the formation of new blood vessels (Arnal et al., 2010). This process, known as angiogenesis, is important in the female reproductive tract physiology as well as in pathophysiological settings such as cardio/cerebrovascular disease, diabetes, endometriosis and cancer, supporting a role for sex hormones in the context of neovascularization process (Losordo and Isner, 2001; Fadini et al., 2008). Estrogen action in the endothelium is mediated by its interaction with estrogen receptor (ER)α and ERβ, which operate as hormone-regulated transcription factors. Membrane ERα and the recently described G protein-coupled estrogen receptor (GPER) also mediate rapid, non-genomic effects (Prossnitz and Barton, 2014). Notably, the ER expression pattern in ECs differs according to the location and vessel type (Franconi et al., 2017; Huxley et al., 2018). Estrogenic agents have been reported to enhance new vessel formation by interacting with ERα (Arnal et al., 2010; Sanchez et al., 2011). We recently showed that estrogen treatment enhances angiogenesis in HUVECs by interacting with GPER resulting in the rapid downstream activation of 6-phosphofructo-2-kinase/fructose-2,6-biphosphatase 3 (PFKFB3), a bifunctional protein linking glucose metabolism to endothelial cell proliferation (Trenti et al., 2017a). However, in the above study endothelial cells were not stratified by donor's sex, nor were sex-specific responses to estrogenic agents assessed.

The major steps of the angiogenetic process including EC migration, i.e. chemotaxis, and the ability to form tube-like structures when seeded onto extracellular matrix components involve the modification and spatial organization of actin cytoskeleton (Madri and Pratt, 1986). Estrogen treatment in HUVECs induces tyrosine phosphorylation and activation of focal adhesion kinase (FAK), a signaling protein located at sites of integrin clustering that controls cell migration (Sanchez et al., 2011). It has been reported that PFK15, a small molecular PFKFB3 inhibitor, causes a decrease in phosphorylated FAK in gastric cancer cells, thereby limiting cell invasiveness *in vitro* (Zhu et al., 2016). We found that digitoxin at clinically relevant concentrations elicits a potent anti-angiogenic effect *in vitro* and *in vivo* by inhibiting FAK activation (Trenti et al., 2017b). However, whether FAK activation and the responses to pharmacological agents affecting angiogenesis are sexually dimorphic is largely unknown.

Translation of research findings into clinical recommendations requires the identification of possible targets and/or mechanisms accounting for, e.g., gender-specific response to pharmacological treatment. Therefore, the aim of this study was to further explore the relative functional role of sex and estrogenic agents in the healthy endothelium using *in vitro* approaches that mimic the main steps of the angiogenic process. Additional specific aims were to unravel potential sex differences in key proteins involved in the migration and tubularization of HUVECs, and to test sex-specific responses to pharmacological treatment *in vitro*.

## Materials and Methods

### Drugs and Chemicals

Cell culture reagents and fetal bovine serum (FBS, cat No. 10270-106) were purchased from Invitrogen (Carlsbad, CA, United States). Endothelial cell growth supplement (ECGS, cat No. E2759), Trypan Blue (cat No. T8154), LY 294002 (Cat No. 154447-36-6), 3-4,5 dimethylthiazol-2-yl-2,5 diphenyltetrazolium bromide (MTT, cat No. M2128) and 17β-estradiol (E2, cat No. E-8875) were from Sigma-Aldrich (St. Louis, MO, United States). Collagen (rat-tail) was from Roche (Basel, Switzerland). (6)-1-(3aR*,4S*,9bS*)-4-(6-bromo-1,3-benzodioxol-5-yl)-3a,4,5,9b-tetrahydro-3H-cyclopenta-cquinolin-8-yl-ethanone (G1, cat No. 3577) was purchased from Tocris Bioscience (Bristol, United Kingdom).

### HUVEC Isolation and Culture

HUVECs were isolated as previously described (Bolego et al., 2006a; Bolego et al., 2006b) from human umbilical cords collected after delivery from full-term normal pregnancies at the Obstetrics and Gynecological Unit of Padua University Hospital. The mothers gave their informed consent, and collected cords were identifiable only by sex. The procedure was approved by Padua University Hospital Ethics Committee. All experiments were performed in accordance with relevant guidelines and regulations. Cells were obtained from *n* = 16 male and *n* = 16 female donors; one cell preparation was derived from each donor. HUVECs were grown at 37°C and 5% CO_2_ in complete medium (M199 supplemented with 15% FBS, 100 μg/ml ECGS, 100 U/mL heparin, 2 mM glutamine, gentamicin (40 μg/ml, cat No. 15710049, Invitrogen). HUVECs were used from passage 2–4, and cells from male and female donors at the same passage were used in each independent experiment. For experiments with estrogenic agents, cells were switched to complete phenol red-free M199 72 h before each assay (because of phenol red weak estrogen activity that can stimulate some estrogen-sensitive cells) and to culture medium with 5% serum overnight before the experiment. The experiments with E2 or G1 were performed using phenol-free M199 medium supplemented with 5% FBS, 40 μg/ml gentamicin, 100 μg/ml ECGS and 100 UI/mL heparin.

### Genotyping

HUVEC sex was determined by analysis of amelogenin gene. DNA from 3 × 10^5^ HUVECs was extracted using the DNA/RNA Mini Kit (Qiagen, Hilden, Germany) following the manufacturer's protocol. 100 ng of genomic DNA was amplified using MyTaq HS Red Mix2X using the following primers: AMELU1 5′-CCC​TTT​GAA​GTG​GTA​CCA​GAG​CA-3′ and AMELD1 5′-GCA​TGC​CTA​ATA​TTT​TCA​GGG​AAT​A-3′ (Mannucci et al., 1994). Samples were amplified through an initial denaturation of 1 min at 95°C following 35 cycles comprising 15 s at 95°C, 15 s at 57°C and 15 s at 72°C. PCR products were then separated on a 6% agarose gel with SYBR Safe DNA Gel Stain (Invitrogen, Carlsbad, CA, United States). Images were acquired with Azure Imaging System.

### MTT Assay

HUVECs were seeded in 96-well plates under different conditions as indicated in Results. The next day, cells were incubated in the presence or absence of E2 or G1 (1–100 nM) with fresh medium for 24–72 h. Four hours before the end of incubation, 10 μL of MTT (5 mg/ml in phosphate-buffered saline (PBS)) was added to each well. Then, the medium was removed, and formazan crystals were dissolved in 100 μL dimethyl sulfoxide. MTT reduction was quantified by measuring light absorbance with a Wallac Victor2 plate reader (PerkinElmer, Waltham, MA, United States) at 570–630 nm. Background absorbance values from control wells (cell-free media) were subtracted. Cell viability is expressed as raw optical density value.

### Trypan Blue Exclusion Assay

HUVECs (8 × 10^4^ cells/well in 35-mm dishes) were plated in complete culture medium. The following day and after 72 h the culture medium was removed and replaced with fresh medium. Cells were harvested at different time points (24–144 h); then Trypan blue was added to the cell suspension to a final concentration of 0.2%. Cells excluding Trypan blue (viable cells) as wells as dead cells were counted under the microscope with a Bürker haemocytometer. Cell proliferation is expressed as number of viable cells.

### Immunocytochemistry

HUVECs (8 × 10^4^ cells/well) were seeded in 12-well plates containing glass coverslips in complete culture medium. The next day, living cells were incubated with 10% FBS in dPBS (PBS containing Ca^2+^ and Mg^2+^) to block unspecific sites. The following antibodies were used: rabbit anti-GPER (1:100, cat No. 39742, Abcam) and mouse anti-CD31 (1:100, Cat No.550389, BD Bioscience) diluted in 10% FBS in dPBS. Secondary antibodies for immunofluorescence (anti-rabbit Alexa 488 (Cat No. A32731) and anti-mouse Alexa 555 (Cat No. A32727), Invitrogen, Carlsbad, CA, United States) were used at 1:200 and 1:300, respectively, in 10% FBS in dPBS. Nuclear staining was carried out with Hoechst stain (1:1,000; Cat No. 33342, Invitrogen). Slides were mounted with Mounting Medium (Cat No. M1289, Sigma-Aldrich) and confocal images were immediately acquired through ×60 objective with an LSM 800 (Zeiss) confocal microscope and analyzed using Zen Blue 2.0. Figure panels were assembled using ImageJ 1,47v (NIH, United States).

### Western Blot

HUVECs (3 × 10^5^ cells) were seeded in 35-mm dishes in complete culture medium. Cells were treated as indicated in Results and lysed with lysis buffer (PBS supplemented with 1.2% Triton X-100 (cat No. 1001016696, Sigma), Roche cOmpleteTM inhibitor cocktail 1X (cat No. 11697498001), 2.5 mM NaF (cat No. S-1504, Sigma), 2 mM sodium pyrophosphate (cat No. S-6422, Sigma), 4 mM Na orthovanadate (cat No. S6508, Sigma), 1 mM PMSF (cat No. P-7626, Sigma)). After centrifugation at 10,000 g for 15 min, supernatants were harvested for SDS-PAGE and Western blotting essentially as described (Trenti et al., 2017a; Trenti et al., 2017b). Protein quantification was performed using the BCA assay (Sigma). Proteins (45 μg) were separated on SDS-PAGE and transferred onto PVDF membranes (Hybond-P, Amersham, Little Chalfont, United Kingdom). Membranes were then blocked and probed using the following primary antibodies: mouse anti-FAK (1:1,000, cat No. 55632), rabbit anti-Src (1:2,000, cat No. 133283), rabbit anti-PFKFB3 (1:5,000, cat No. 181861), rabbit anti-GPER (1:500, cat No. 39742) and rabbit anti-GAPDH (1:10,000, cat No. 181602); all from Abcam, Cambridge, United Kingdom), rabbit anti-phospho-FAK (Y576/577) (1:500 dilution, cat No. 3281S), rabbit anti-phospho-Src (Y416) (1:1,000, cat No. 2101S), rabbit anti-phospho-Akt (S473) (1:500, cat No. 9271), rabbit anti-Akt (1:500, cat No. 9272); Cell Signaling Technology, Danvers, MA, United States). After washing, membranes were incubated with appropriate secondary HRP-conjugated antibodies (Vector Laboratories, cat No. PI-1000 and PI-2000, Burlingame, California; United States) at 1:10,000 dilution. Bands were detected by chemiluminescence using the LiteAblot Turbo (Euroclone, Pero, Italy). Images were acquired with Azure Imaging System (Azure Biosystem, Dublin, CA, United States). Densitometric analysis of bands was performed with ImageJ 1,47v (NIH, United States). Data are expressed as relative protein levels with respect to the loading control GAPDH.

### Chemotaxis Assay

Chemotaxis experiments were performed in a 48-well modified microchemotaxis chamber (Neuro Probe, Gaithersburg, MD, United States) using 8 μm nucleoporepolyvinylpyrrolidine-free polycarbonate filters coated with 10 μg/ml collagen. Lower chambers were filled with M199 supplemented with 100 U/mL heparin and 5% or 15% FBS. Upper chambers were filled with 50 μL HUVEC suspension (1.6 × 10^5^ cells/mL in M199 supplemented with 1% FBS and 100 U/mL heparin). Inhibitors were added in both the upper and lower compartment. For assessment of basal motility, M199 supplemented with 1% FBS, 100 U/mL heparin was added in the lower chamber. After 6 h incubation at 37°C, non-migrating cells on the upper filter surface were removed by scraping. The cells migrated to the lower side of the filter were stained with Diff-Quick stain (VWR Scientific Products, Bridgeport, NJ, United States), and densitometric analysis was performed using the ImageJ 1,47v software (NIH, United States). Each experiment was performed in sextuplicate. Results are reported as arbitrary units of optical density.

### Capillary–like Tube Formation Assay

HUVECs (8×10^3^ cells/well) were plated onto a thin layer (50 µL) of basement membrane matrix (Matrigel™, cat No. 354234, Corning Corp., Corning, NY, United States) in 96-well plates and incubated at 37°C for 4 h in complete culture medium. One image per well was captured at 4X under a bright field inverted microscope (Nikon Eclipse Ti, Shinagawa, Tokyo, Japan) equipped with a digital camera. Images were analysed using Angiogenesis Analyzer, a plugin developed for the ImageJ software (Carpentier et al., 2012). Data on topological parameters (number of junctions, master segments, meshes and total mesh area) of the capillary-like network were analyzed in each well. Data are expressed as absolute values.

### Statistical Analysis

Results are presented as mean values from individual cell preparations obtained from each donor, with error bars representing the standard error of the mean (SEM) value. Statistical analysis was performed using GraphPad Prism 5.02 (GraphPad Software Inc., La Jolla, CA, United States). Student's t-test was used to compare means of two independent groups, whereas two-way analysis of variance (ANOVA) was used to compare gender differences in cell viability over time and in the concentration-response curve to digitoxin (Ludbrook, 1994). A *p* value of <0.05 was considered statistically significant.

## Results

### Sex Chromosomes but not Estrogenic Agents Affected HUVEC Viability and Proliferation

**FIGURE 1:**
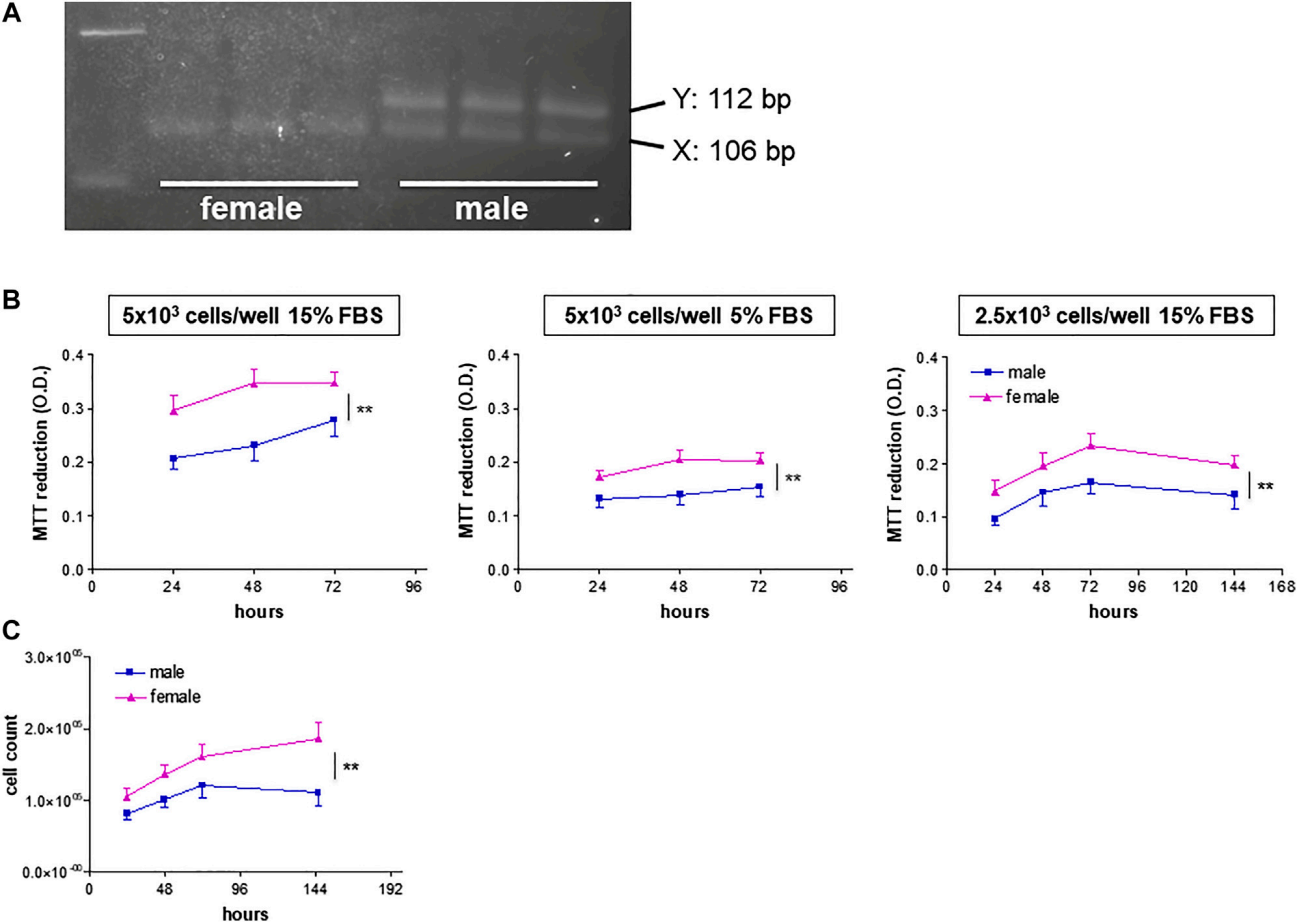
Genotyping and growth rate of HUVECs from male and female donors. **(A)** Representative agarose gels of amelogenin PCR products from HUVEC DNA samples of female **(left)** and male **(right)** donors. **(B)**. HUVECs were plated in 96-well plates in M199 medium supplemented with ECGS (0.1 mg/ml) and heparin (100 IU/ml) at the indicated FBS concentrations and seeding densities. Cell viability is shown as optical density (O.D.) values (MTT assays). Each independent experiment (*n* = 4) was performed in triplicate using cells from three female and three male donors. Data represent mean ± SEM of 12 donors; two-way ANOVA for comparison between curves, **p < 0.01. **(C)**. HUVECs (8 × 10^4^ cells/well) were plated in 6-well plates in M199 medium supplemented with 15% FBS, ECGS (0.1 mg/ml) and heparin (100 UI/mL). Proliferation rates are expressed as vital cell counts after Trypan blue staining at indicated time points. Each independent experiment (*n* = 3) was performed in duplicate using cells from two female and two male individual donors. Data represent mean ± SEM of six donors; two-way ANOVA for comparison between curves, **p < 0.01.

The HUVEC donors’ sex was confirmed by amelogenin DNA amplification. AmelX and AmelY amplicons had different size (106 and 112 bp, respectively), thereby allowing separation on agarose gels (Figure 1A).

We then tested cell viability as measured by MTT assay under a variety of culture conditions. When grown at high cell density (5 × 10^3^ cells/well) in M199 medium supplemented with 15% FBS and ECGS, HUVECs from female donors (fHUVECs) showed higher viability than those of male donors (mHUVECs) over a 3-days time course (Figure 1B, left panel; *n* = 4). This was also observed when FBS supplementation was reduced to 5% (Figure 1B, middle panel), a condition slowing down cell proliferation that was selected to test the effects of estrogenic agents. fHUVEC viability also remained higher than that of mHUVECs at lower baseline cell density (2.5 × 10^3^ cells/well; Figure 1B, right panel), which allowed longer culture for up to 6 days. As the MTT system does not discriminate between increased proliferation and reduced death rate of cells, proliferation was specifically tested by cell counting and Trypan blue staining over 6 days under optimal culture conditions. Again, higher counts were observed for vital fHUVECs than for mHUVECs (Figure 1C), with no sex-differences in the percentage of dead cells (about 5% at 1 week). This is in line with previous proliferation rate findings under high-serum culture conditions (Addis et al., 2014).

**FIGURE 2:**
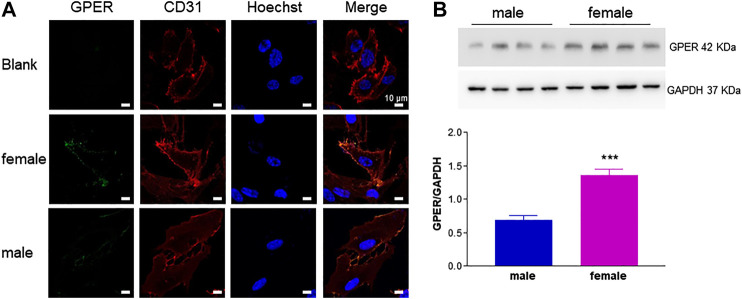
GPER detection in HUVECs from female and male donors. **A**. HUVECs were incubated with anti-GPER and anti-CD31 antibodies. Nuclei were labeled with Hoechst stain. Images from left to right show subcellular localization of GPER (green), CD31 (red) and cell nuclei (blue), respectively. Blank sample was incubated only with CD31 primary antibody, but with both (green and red) secondary antibodies. Representative confocal images (600×); scale bar 10 μm. **(B)**. GPER immunodetection. A representative experiment is shown in the upper panel. GAPDH expression was used as loading control. Bands from independent experiments (*n* = 2) performed using cells from four male and four female donors were quantified by densitometric analysis and normalized to GAPDH (mean ± SEM of 8 male and 8 female donors). *t*-test; ***p < 0.001.

**FIGURE 3:**
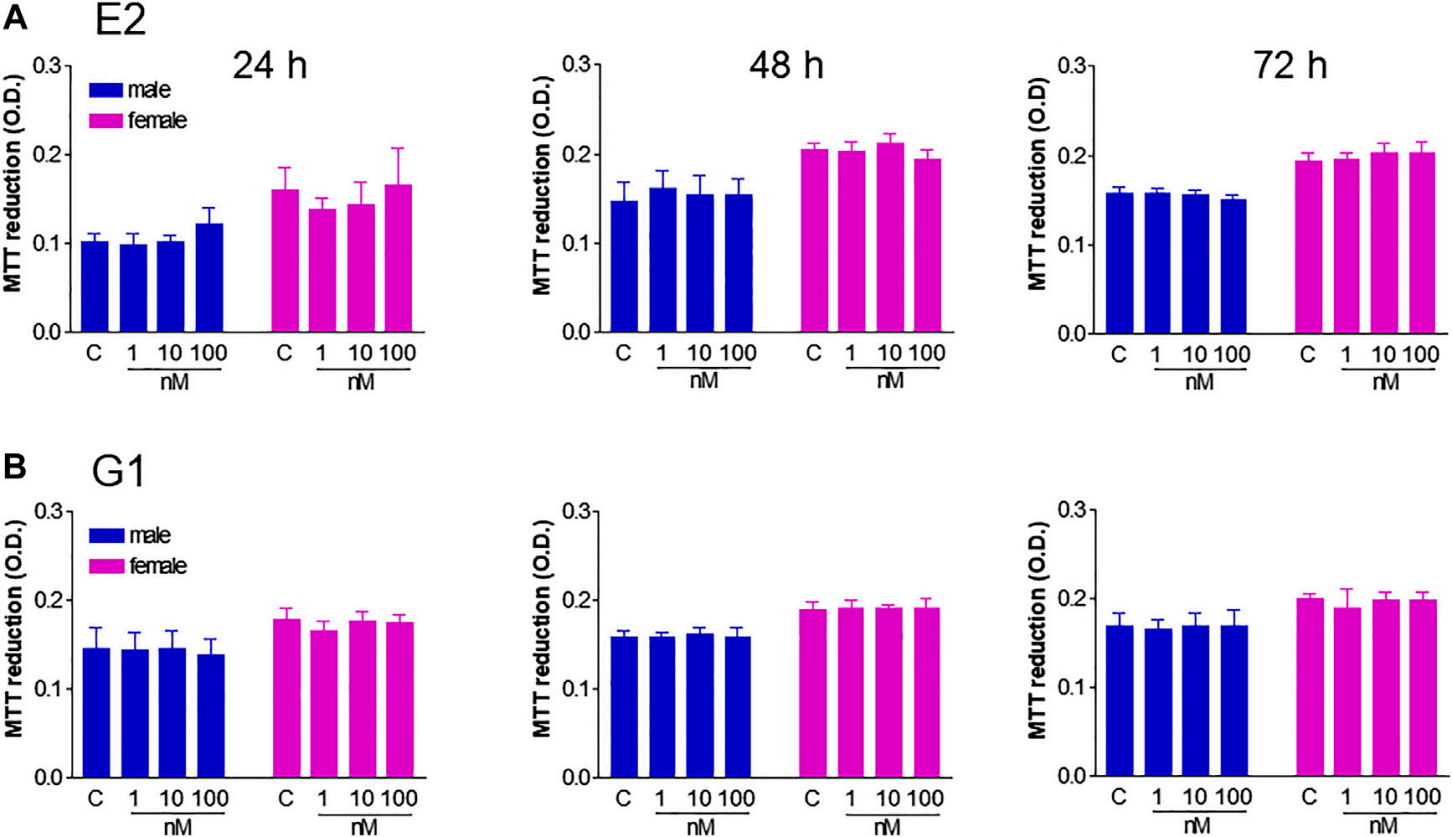
Effect of E2 and G1 on HUVEC viability. HUVECs from male (blue) and female (pink) donors (5 × 10^3^ cells/well) were grown in phenol red-free M199 medium supplemented with 5% FBS in the presence of ECGS (0.1 mg/ml) and heparin (100 UI/mL) until confluency, and treated with E2 (1–100 nM, panel **A)**, G1 (1–100 nM, panel **B)** or vehicle (C) for 24–72 h. Cell viability is shown as absolute optical density (O.D.) values (MTT assays). Each independent experiment (*n* = 4) was performed in triplicate using cells from 1 male and 1 female donor. Data represent mean ± SEM. *t*-test vs. control; ns.

Next, we investigated the relative contribution of estrogenic agents to the sexually dimorphic HUVEC growth rate. While ERs are known to be expressed in HUVECs, evidence regarding gender specificity in ER expression is controversial (Addis et al., 2014; Kim-Schulze et al., 1996; Chakrabarti and Davidge, 2012). We here provide novel evidence for GPER membrane localization by confocal microscopy (Figure 2A) and confirm that it was more abundant in fHUVECs than in mHUVECs by Western blot (Figure 2B; Addis et al., 2014). In view of such differential receptor expression pattern, and because previous studies focused only on the effects of physiological estrogen, HUVECs of both sexes (5 × 10^3^ cells/well) were treated with the nonselective ER agonist E2 (Figure 3A) or the selective GPER agonist G1 (Figure 3B; both 1–100 nM) for 1–3 days. None of the above treatments affected cell viability with respect to control as measured by MTT assays. In addition, no gender differences were observed in response to E2/G1 treatment. Such unresponsiveness was observed irrespective of cell density at baseline, % FBS, treatment duration and ECGS or heparin supplementation. This is in contrast with previous reports showing increased proliferation of E2-treated cells, suggesting that estrogenic agents are also likely to induce an anti-apoptotic effect under different culture conditions (Morales et al., 1995).

Overall, our data suggest that sex more than estrogenic agents accounted for increased growth rate of fHUVECs vs. mHUVECs.

### Increased *in vitro* Angiogenic Potential of fHUVECs Under Physiological Conditions

**FIGURE 4:**
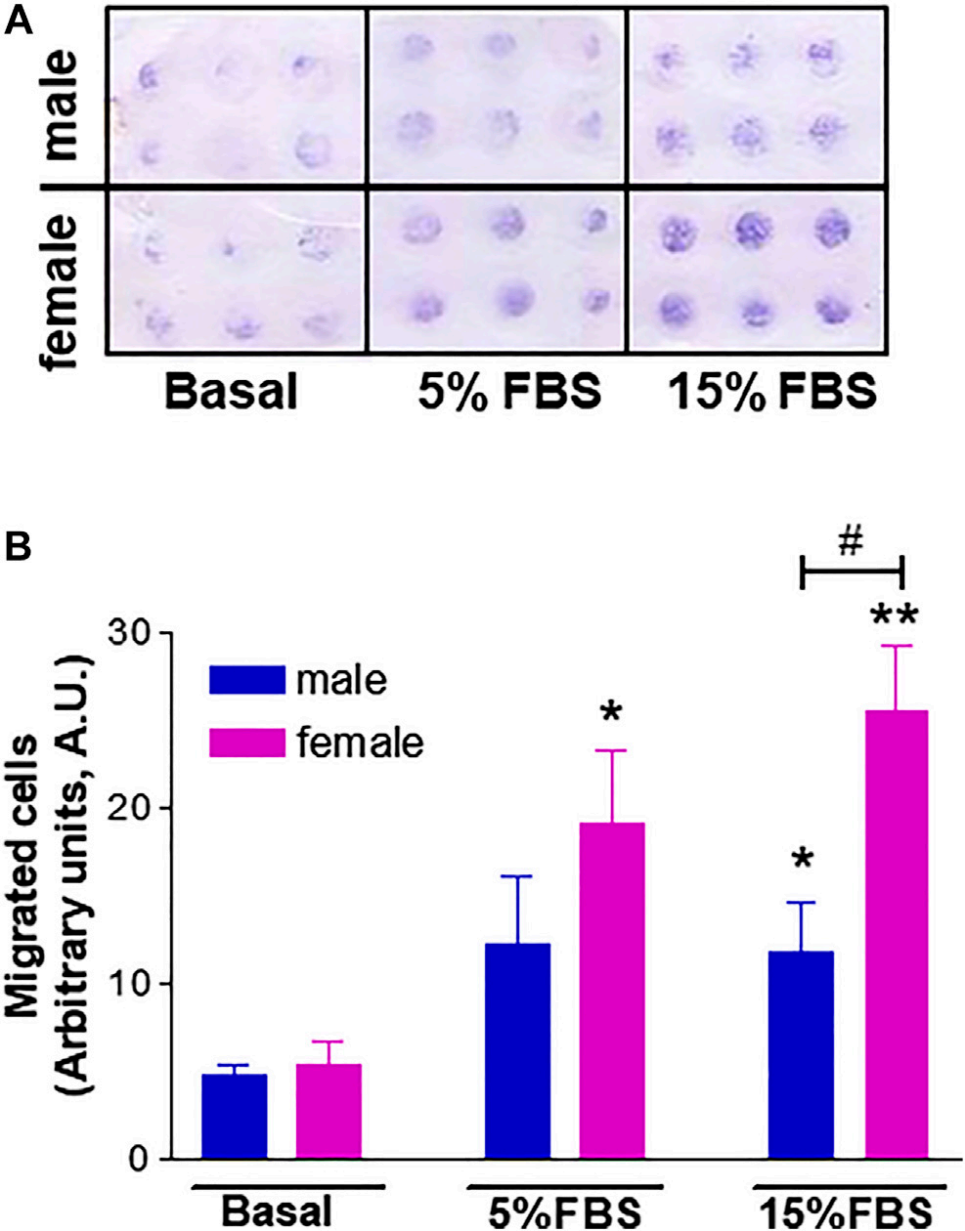
Effect of donor's sex on HUVEC migration in a chemotaxis assay using 5% or 15% FBS as a chemoattractant agent. **(A)**. Representative image of HUVEC migration in a modified 48-well Boyden chamber after 6 h incubation at 37°C. **(B)**. Cell migration is shown as optical density values (A.U., arbitrary units). Each independent experiment (*n* = 4) was performed in sextuplicate using cells from one male and one female donor. Data are expressed as mean ± SEM. *t*-test; *p < 0.05, **p < 0.01 (vs. basal); ^#^ *p* < 0.05.

**FIGURE 5:**
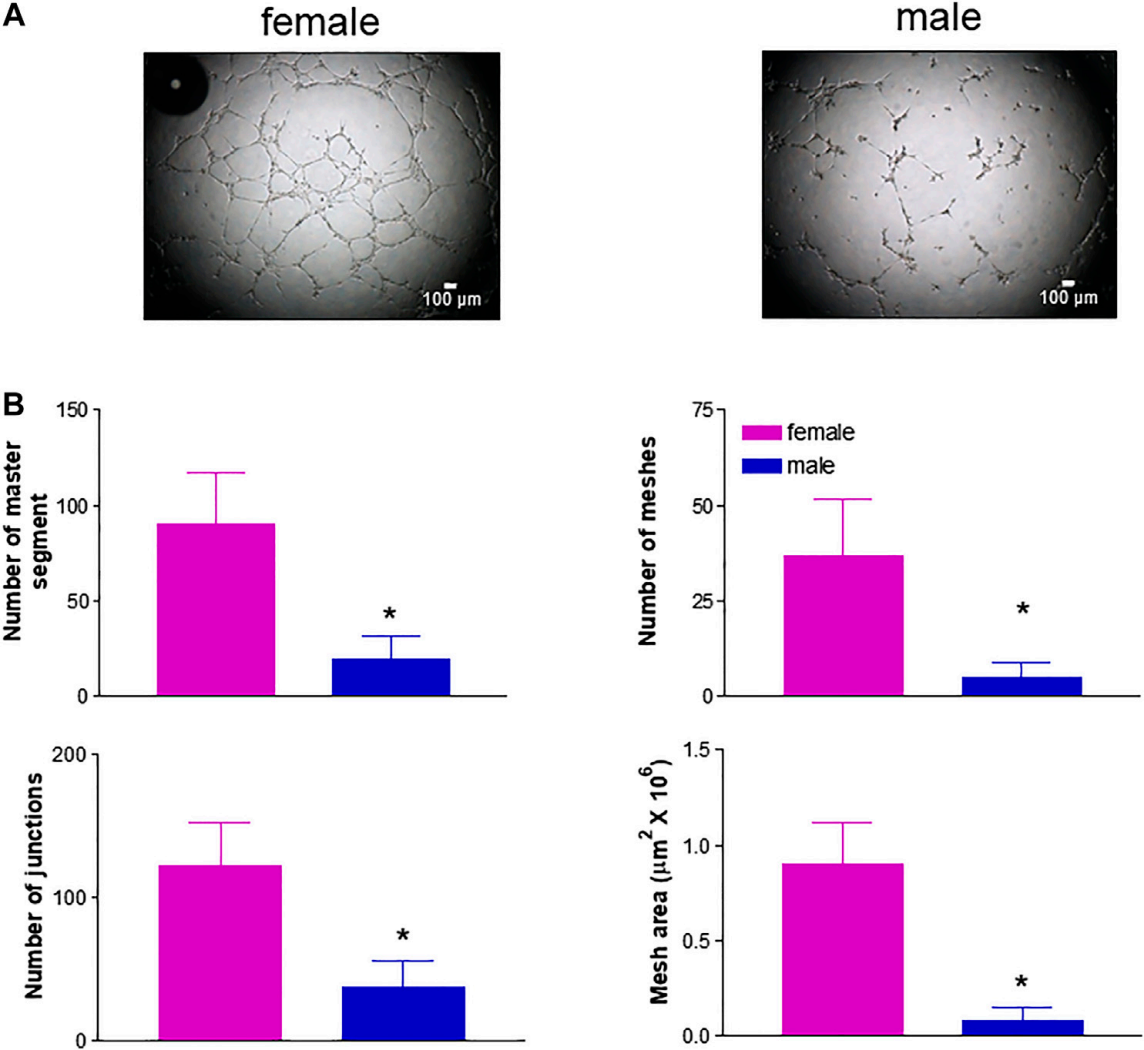
Effect of donor's sex on capillary tube-like formation. HUVECs were seeded onto 96-well plates coated with Matrigel in complete culture medium. After incubating for 4 h one micrograph image per well was taken. **(A)**. Images from a representative experiment; scale bar: 100 μm. **(B)**. Analysis of specific parameters of capillary tube formation measured using Angiogenesis Analyser (ImageJ). Each independent experiment (*n* = 3) was performed using cells from 1 male and 1 female donor. Data are expressed as mean ± SEM. *t*-test, *p < 0.05.

Based on the knowledge that neovascularization is a physiological feature in the female reproductive tract and is regulated by estrogenic agents (Rubanyi et al., 2002; Trenti et al., 2017a), we further explored potential gender differences using a number of additional *in vitro* experimental approaches that mimic the major steps of the angiogenic process (Simons et al., 2015). We first performed a chemotaxis assay to assess HUVEC migration in response to 5% and 15% FBS using a well-established cell migration technique, i.e. the microchemotaxis assay, and found increased migration of fHUVECs with respect to mHUVECs (Figure 4). HUVEC collective bi-dimensional migration (not stimulated by soluble chemoattractants) tested through the wound healing assay was also increased in fHUVECs with respect to mHUVECs (Supplementary Figure S1). Tubularization is the process of organization of HUVECs into capillary tube-like structures when cultured onto extracellular matrix proteins (Matrigel assay) that recapitulates several steps of the angiogenic process *in vivo*. Sexual dimorphism was also detectable in this assay: in fact, topological parameters (number of junctions, master segments, meshes and total mesh area) of the capillary-like network were markedly enhanced in fHUVECs with respect to mHUVECs under standard culture conditions (Figure 5). Overall, these results indicate that fHUVECs display enhanced *in vitro* angiogenic potential compared with mHUVECs.

### Female Hormones and HUVEC Donors’ Sex Specifically Affect the Activity of Key Angiogenic Proteins

**FIGURE 6:**
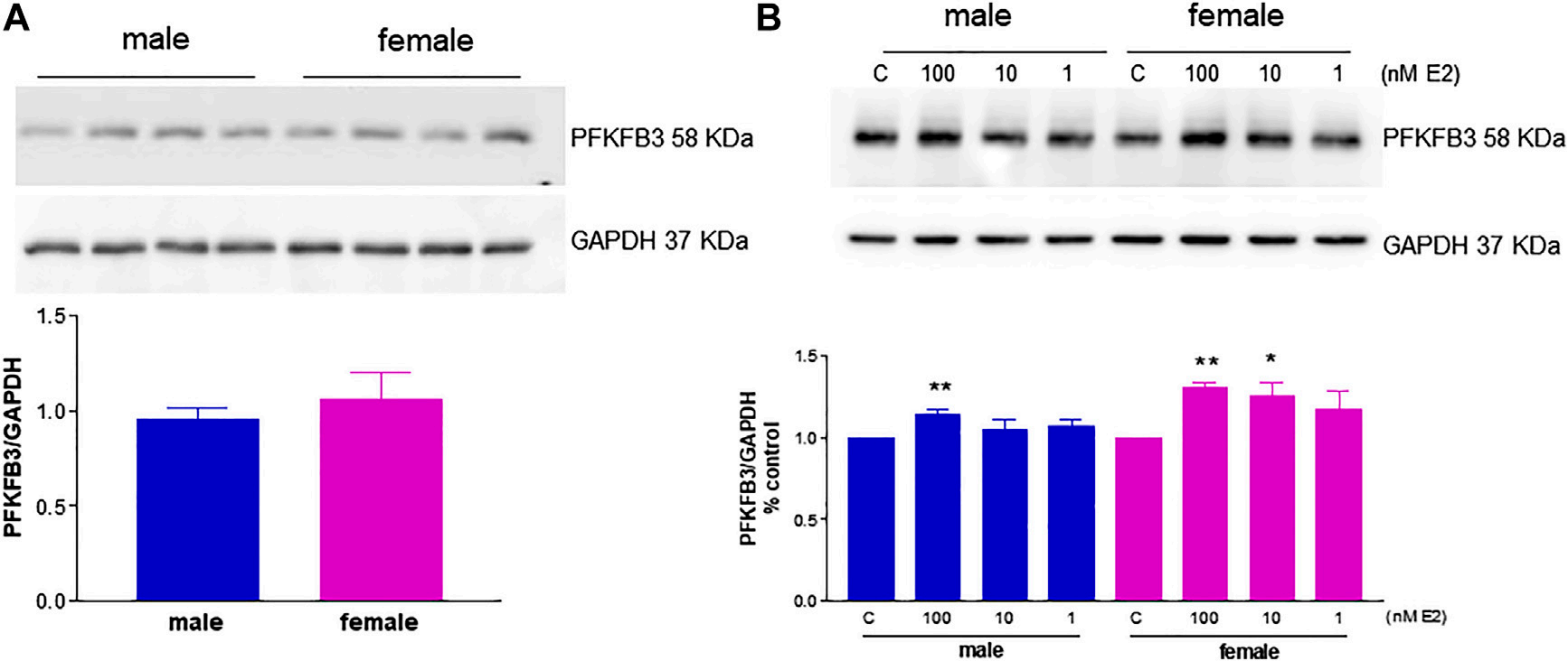
Immunodetection of the glycolytic activator PFKFB3 in HUVECs from male and female donors in the presence or absence of E2. **(A)**. HUVECs were seeded in 35-mm dishes in M199 complete medium. ***Upper panel*:** Representative western blot showing baseline PFKFB3 immunodetection; GAPDH expression was used as a loading control. ***Lower panel*** **:** Densitometric analysis of bands, normalized to GAPDH levels. Each independent experiment (*n* = 3) was performed using cells from 3–4 male and 3–4 female donors. Data are expressed as the mean ± SEM of 11 male and 11 female donors; *t*-test, ns. **(B)**. HUVECs were seeded in 35-mm dishes in M199 phenol red–free medium with 5% FBS and treated with 1–100 nM E2 or vehicle for 3 h. ***Upper panel*** **:** Representative Western blot showing PFKFB3 immunodetection; GAPDH expression was used as a loading control. C, control. ***Lower panel*:** Densitometric analysis of bands, normalized to GADPH levels. Values in the C group were set as 1. Each independent experiment (*n* = 4) was performed using cells from one male and one female donor. Data are expressed as the mean ± SEM. *t*-test, *p < 0.05; **p < 0.01 *vs*. **C**.

In the setting of the angiogenic process, HUVEC functional phenotypes are largely determined by enzymes involved in glucose metabolism and migratory properties such as the glycolytic enzyme activator PFKFB3 and FAK, both of which are emerging drug targets for angiogenesis (Schoors et al., 2014; Lee et al., 2015). In particular, PFKFB3 inhibition results in impaired pathological angiogenesis (Schoors et al., 2014), and we previously reported that PFKFB3 silencing reduces estrogen-mediated angiogenesis (Trenti et al., 2017a). To test a possible role for this enzyme in gender differences in the angiogenic process, we treated the cells with the PFKFB3 inhibitor 3PO and found that FBS-induced migration was blocked in both fHUVECs and mHUVECs (Supplementary Figure S2). PFKFB3 levels did not significantly differ between fHUVECs and mHUVECs (Figure 6A). In order to assess possible gender differences in estrogenic responses, fHUVECs and mHUVECs were treated with increasing concentrations of E2 (1-100 nM). PFKFB3 protein levels were enhanced by E2 with higher potency in fHUVECs vs. mHUVECs. Indeed, 10 nM E2 significantly increased PFKFB3 levels in fHUVECs only (Figure 6B). Overall, these findings point to sex hormones as more critical regulators of this metabolic enzyme with respect to sex chromosomes.

**FIGURE 7:**
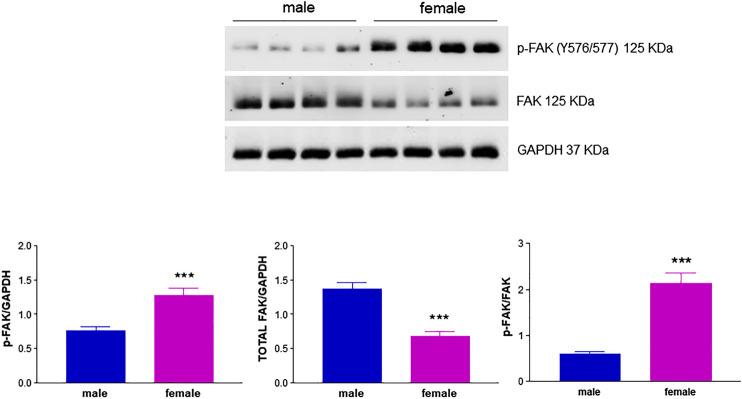
Effect of HUVEC donor's sex on FAK activation. HUVECs were seeded in 35-mm dishes in M199 complete medium. ***Upper panel*** **:** Representative Western blots showing the expression of p-FAK (Y576/577) and total FAK; GAPDH was used as loading control. ***Lower panel*** **:** Densitometric analysis of bands normalized to GAPDH levels and p-FAK/FAK ratio. Each independent experiment (*n* = 4) was performed using cells from four male and four female donors. Data are shown as the mean ± SEM of 16 male and 16 female donors. *t*-test, ***p < 0.001.

**FIGURE 8:**
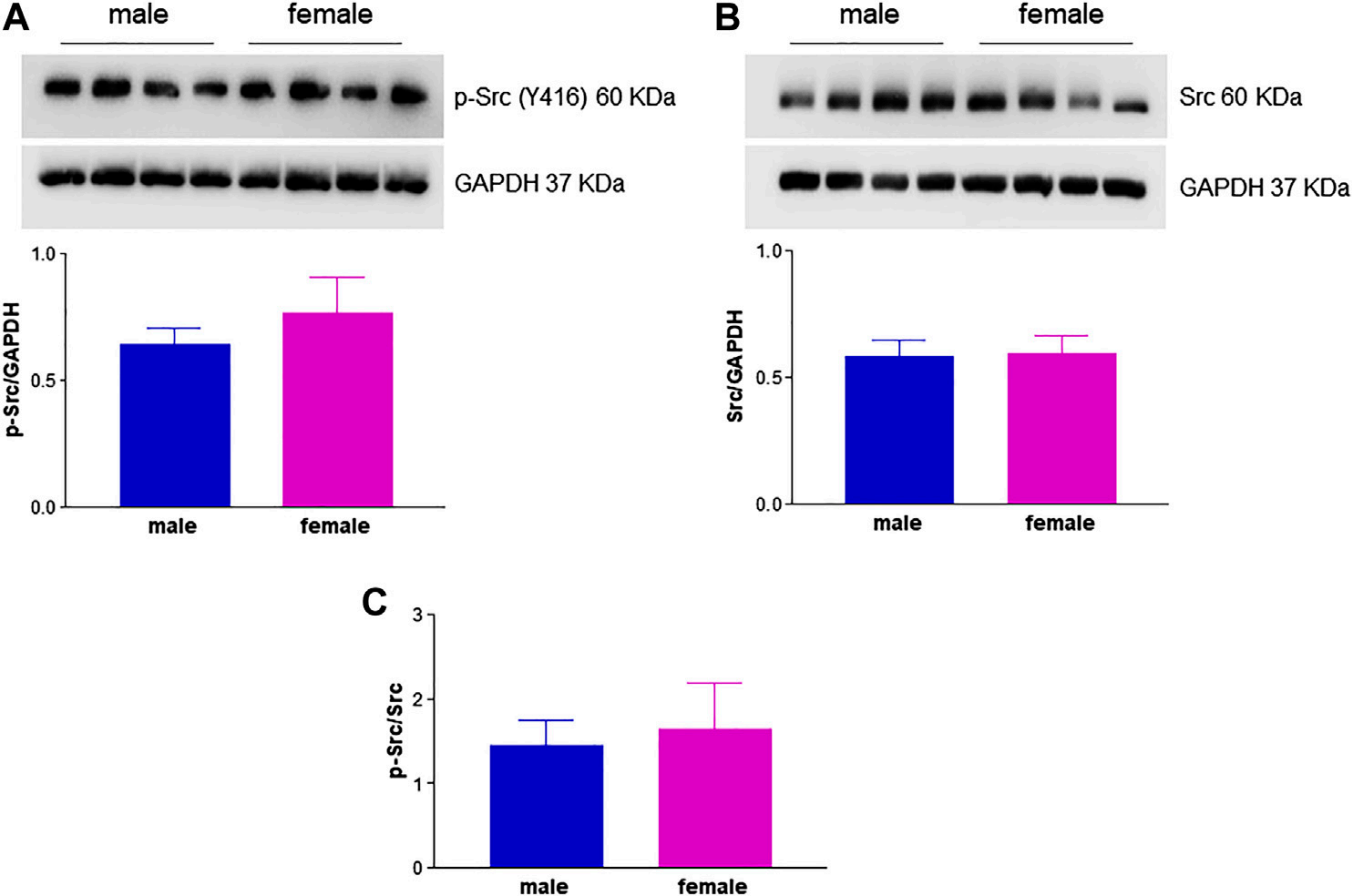
Effect of HUVEC donor's sex on Src activation. HUVECs were seeded in 35-mm dishes in M199 complete medium. **(A,B)** ***Upper panels*** **:** Representative Western blots showing the expression of p-Src (Y416) and total Src, respectively; GAPDH was used as loading control. ***Lower panel*** **:** Densitometric analysis of bands normalized to GAPDH levels. **(C)** p-Src/Src ratio. Each independent experiment (*n* = 3) was performed using cells from four male and four female donors. Data are shown as mean ± SEM of 12 male and 12 female donors. *t*-test, ns.

**FIGURE 9:**
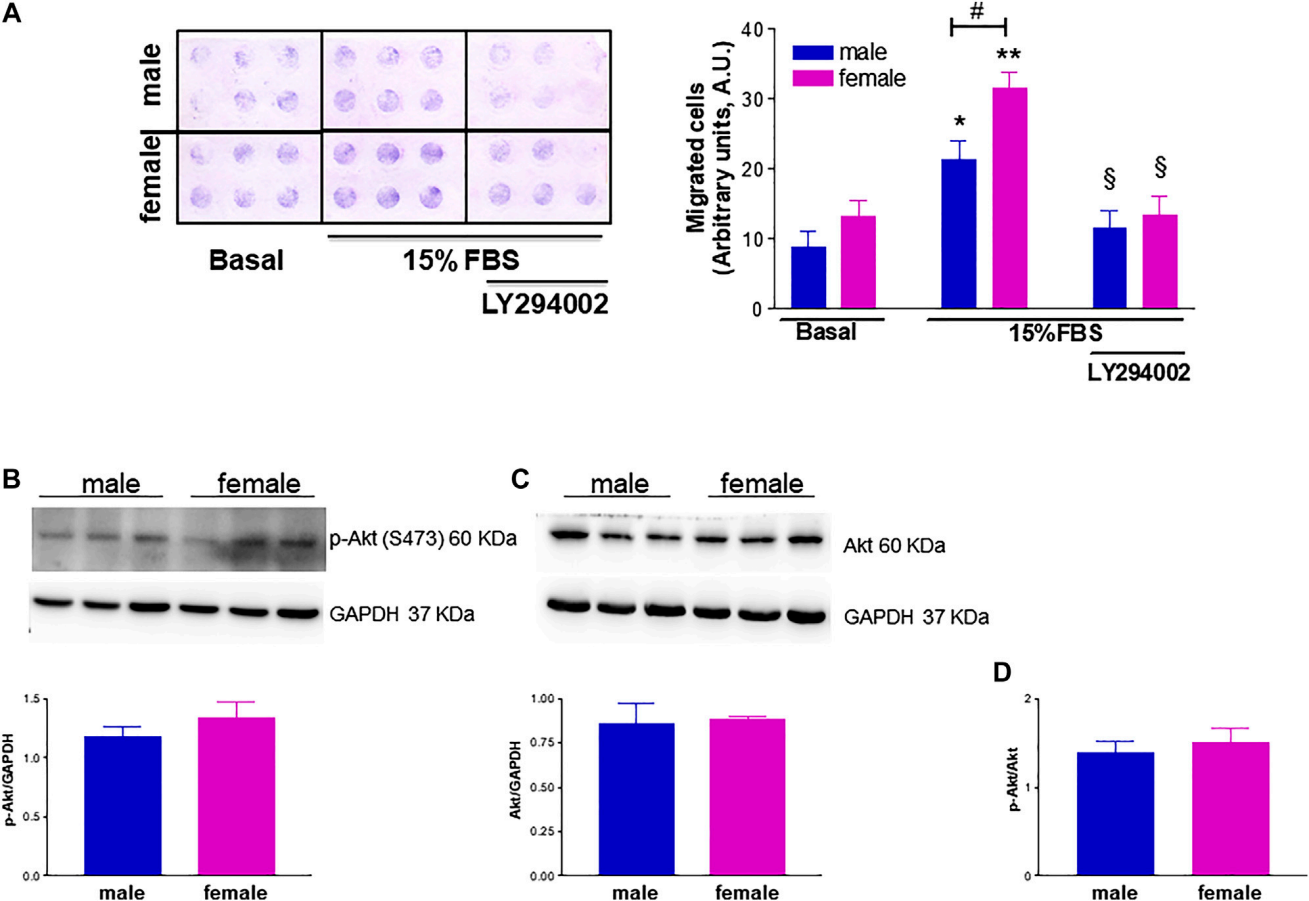
Effect of phosphatidylinositol 3-kinase (PI3K) inhibition on FBS-induced HUVEC migration and effect of donor's sex on Akt activation. **(A)**. The PI3K inhibitor LY294002 (25 µM) was tested in a chemotaxis assay on HUVECs from male and female donors using 15% FBS as a chemoattractant agent. ***Left panel:*** Representative image of HUVEC migration in a modified 48-well Boyden chamber after 6 h incubation at 37°C. ***Right panel:*** Cell migration is shown as optical density values (A.U., arbitrary units). Each independent experiment (*n* = 4) was performed in sextuplicate using cells from one male and one female donor. Data are expressed as mean ± SEM. *t*-test, *p < 0.05, **p < 0.01 (vs. basal); ^#^ *p* < 0.05; §*p* < 0.05 (vs. 15% FBS alone). **(B, C).** HUVECs were seeded in 35-mm dishes in M199 complete medium. ***Upper panels:*** Representative Western blots showing the expression of p-Akt (S473) and total Akt, respectively; GAPDH was used as loading control. ***Lower panels:*** Densitometric analysis of bands normalized to GAPDH levels. Data are shown as mean ± SEM of three male and three female donors. **(D)**. p-Akt/Akt ratio. *t*-test, ns.

Another important mediator of angiogenesis known to be activated by estrogenic agents is endothelial FAK (Sanchez et al., 2011; Rigiracciolo et al., 2019). This non-receptor tyrosine kinase promotes cell migration by integrating signals from extracellular matrix and growth factors (Sieg et al., 2000). Tyrosine phosphorylation by Src family kinases regulates FAK activation, with FAK phosphorylation at Y576/577 showing maximal enzymatic activity (Zhao and Guan, 2011). Remarkably, levels of phospho-FAK Y576/577 were significantly higher in fHUVECs whereas the opposite was true for total FAK levels, leading to a 4-fold increase in the phospho-FAK/total FAK ratio in fHUVECs with respect to mHUVECs (Figure 7). This pattern was not due to differences in Src activation, which was comparable in the two groups (Figure 8). Interestingly, levels of Y576/577-phosphorylated FAK as well as total FAK were significantly decreased by the PFKFB3 inhibitor 3PO (Supplementary Figure S3). We further explored the PI3K/Akt pathway, which has been linked to FAK activation (Zhao and Guan, 2011). In line with the relevant role of this pathway in angiogenesis, the PI3K inhibitor LY294002 abrogated the migration of fHUVECs and mHUVECs (Figure 9A). However, no gender differences were observed in Akt phosphorylation at S473 and the p-Akt/Akt ratio (Figure 9B-D). Overall, these data highlight the key role of FAK activation consistent with the higher potential of fHUVECs to migrate and give rise to capillary tube-like formation with respect to mHUVECs.

### Sex-Specific Response to Drug Treatment

**FIGURE 10:**
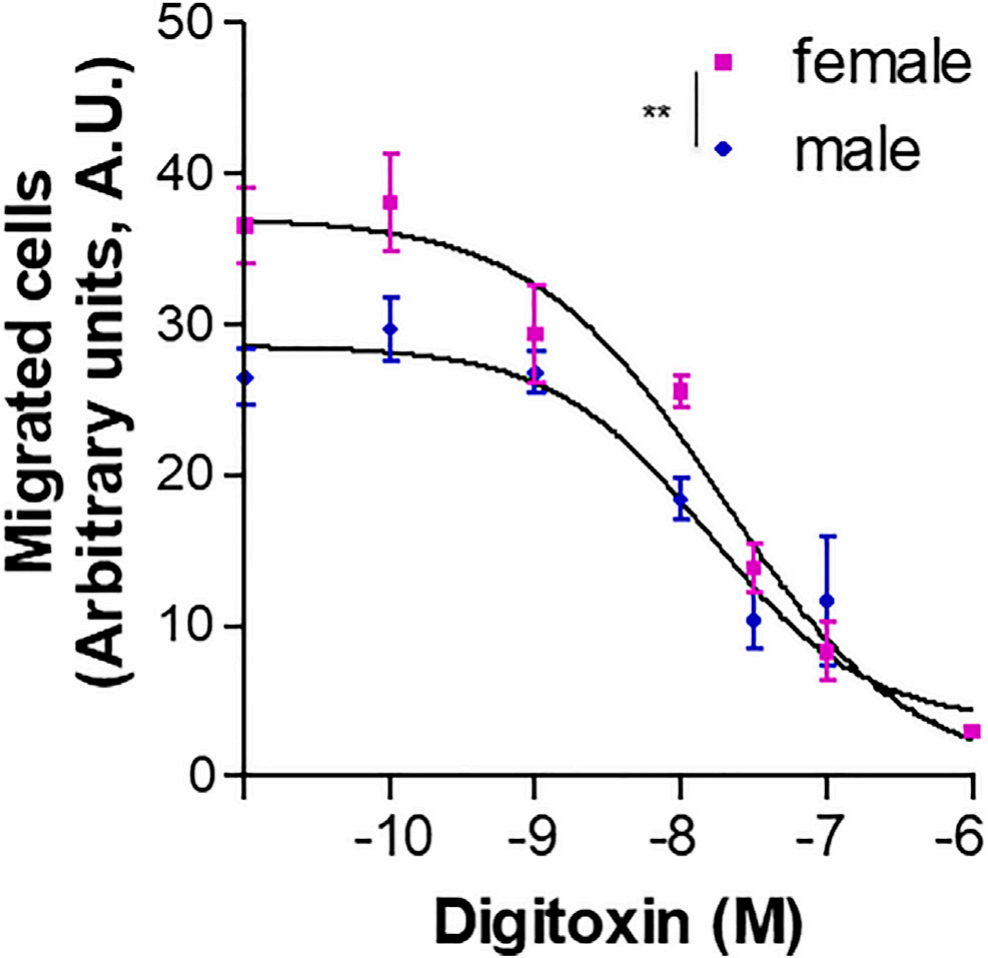
Effect of digitoxin on HUVEC chemotaxis. Migration of HUVECs from male and female donors towards 15% FBS plus 100 μg/ml ECGS was measured in a microchemotaxis chamber in the presence of increasing concentrations (0.1 nM–1 µM) of digitoxin. Cell migration is shown as optical density values (A.U., arbitrary units). Each independent experiment (*n* = 5) was performed in sextuplicate using cells from one male and one female donor. Data are expressed as mean ± SEM. Two-way ANOVA for comparison between curves, **p < 0.01.

We previously reported that the cardiac glycoside digitoxin inhibits HUVEC migration, capillary tube-like formation and *in vivo* angiogenesis by inhibiting growth factor-induced FAK phosphorylation at Y576/577 (Trenti et al., 2017b). Based on this observation, a proof-of-concept assay was performed to test possible gender differences in the pharmacological response to antiangiogenic drugs. Migration of fHUVECs and mHUVECs induced by 15% FBS was measured in a microchemotaxis chamber in the presence of increasing digitoxin concentrations (0.1–100 nM). The concentration-response curves of fHUVEC and mHUVEC were statistically different; nevertheless, IC_50_ values were comparable (fHUVEC: 18.6 nM; mHUVEC: 15.4 nM, respectively; Figure 10). The baseline sexual dimorphism in HUVEC migration was abolished by digitoxin treatment, suggesting that digitoxin at therapeutic concentrations (10–40 nM) is more effective in fHUVECs as compared with mHUVECs, consistent with the increased phospho-FAK Y576/577 levels as observed in fHUVECs (Figure 7).

## Discussion

Gender differences in the cardiovascular system have been largely ascribed to beneficial effects of estrogen on endothelial function. This view has been challenged not only by the mixed outcome of hormone intervention trials but also by emerging evidence of inherent sex differences in endothelial biology (Mudrovcic et al., 2017; Stanhewicz et al., 2018). We herein provide evidence that sex (ECs being XX or XY) and estrogenic agents affect HUVEC phenotypes by complementary modulation of key functional proteins. Notably, consistent with higher pro-angiogenic response in fHUVECs, we herein report sex-specific activation patterns of FAK, a signaling protein involved in cell migration and angiogenesis, as well as of PFKFB3, a master regulator of endothelial glucose metabolism in health and disease.

HUVECs, an established model to investigate endothelial pathophysiology, are suitable to determine the relative contribution of sex at the transcriptional and functional level because the umbilical cord vein is a foetal annex and maintains the foetus sex (Annibalini et al., 2014). In addition, sex differences in freshly isolated HUVECs are independent of maternal estrogen exposure (Hickey et al., 2014). Having assessed unequivocally the donor's sex at the genomic level, we used a combination of MTT assay and Trypan blue staining to measure viable cell numbers and cell proliferation rates, respectively. As a result, fHUVECs were more viable at different cell densities and serum concentrations and had a higher proliferation rate than mHUVECs. One study previously reported higher proliferation rate in fHUVECs than in mHUVECs but no difference in viability (Addis et al., 2014), whereas Cattaneo et al. (2017) reported no sex difference in the proliferation rate. These conflicting findings are likely due to discrepancies in experimental design and procedures compared with ours. In addition, those studies did not compare responses to estrogenic agents in mHUVECs vs fHUVECs.

Endothelial cell growth is known to be stimulated by estrogen treatment *in vitro* (Morales et al., 1995; Kim-Schulze et al., 1996; Geraldes et al., 2002; Oviedo et al., 2007), but sex differences in these responses have not been explored. Functional expression of ERα and ERβ in the endothelium is widely documented (Kim-Schulze et al., 1996; Rubanyi et al., 2002; Addis et al., 2014; Annibalini et al., 2014), with variable patterns according to vessel type (Simard et al., 2011) and donor's sex (Addis et al., 2014; Annibalini et al., 2014). In contrast to previous reports of nuclear GPER staining (Chakrabarti and Davidge, 2012), we herein first provide evidence for plasma membrane localization of GPER in mHUVECs and fHUVECs, with higher expression levels in the latter. Therefore, we tested the hypothesis that a GPER agonist and in parallel the nonselective ER agonist E2 would enhance HUVEC viability in fHUVECs more than in mHUVECs. Yet, treatment with increasing concentrations of E2 and G1 failed to enhance HUVEC viability, regardless of donors' sex. Previous work showed that the GPER agonist G1 triggers the growth rate of bovine vascular ECs (Xu et al., 2017), while others reported attenuated proliferation of the mouse brain microvascular EC line bEnd.3 (Holm et al., 2011) in response to G1, suggesting that EC origin affects endothelial function study outcomes (Huxley et al., 2018). Of note, FBS in our study did not undergo charcoal treatment as in other studies showing estrogen-induced EC proliferation (Morales et al., 1995; Kim-Schulze et al., 1996; Oviedo et al., 2007) because this procedure turns out to be associated with batch-to-batch variation and to remove nutrients essential for cell growth (Cao et al., 2009), thereby impairing HUVEC function including, e.g., sprouting ability (Vanetti et al., 2016). Hence, it is conceivable that increased proliferation rates detected in experiments using charcoal-treated serum (e.g. Morales et al., 1995) resulted at least in part from an anti-apoptotic effect of estrogen (Spyridopoulos et al., 1997; Su et al., 2017). Alternatively, serum supplementation in the culture medium may have provided enough estrogen level to yield a steady growth rate. It would be worthwhile testing the gender-specific effects of other steroid sex hormones on HUVEC growth and proliferation in future studies.

Among other direct effects on resident vascular cells, estrogen fosters endothelial healing also through angiogenesis. This process occurs physiologically in the female reproductive system (Reynolds et al., 1992) and favors neovascularization processes in the context of, e.g., tissue ischemia. Estrogen has been known to promote angiogenesis mainly by interacting with ERα (Losordo and Isner, 2001; Arnal et al., 2010; Sanchez et al., 2011). More recently, we reported that the angiogenic response to estradiol occurs, at least in part, through interaction with GPER, resulting in up-regulation of the glycolytic activator PFKFB3 (Trenti et al., 2017a). However, the relative role of sex and estrogen in angiogenesis has not been completely defined.

Using *in vitro* experimental approaches that mimic the major steps of the angiogenic process we found that, along with increased growth rate, fHUVECs displayed higher migratory properties than mHUVECs in response to 15% and 5% FBS as chemoattractant using the Boyden assay, consistent with previous findings generated using different time points, assays or chemotactic agents (Addis et al., 2014; Cattaneo et al., 2017). However, we here show for the first time that capillary-like tube formation was enhanced in fHUVECs with respect to mHUVECs under physiological conditions. Thus, while comparable observations were made previously in HUVECs exposed to either hyperoxia (Zhang and Lingappan, 2017) or serum starvation (Lorenz et al., 2015), sexual dimorphism in the formation of capillary-like structures occurs and may be relevant under conditions such as development (neovasculogenesis) or physical exercise. Overall, these data suggest a sex-specific chromosome-based control of ECs under basal conditions and in response to environmental cues triggering proliferation, migration and tubulogenesis that may be independent from the contribution of female hormones, at least in the present *in vitro* setting. It is conceivable that sex-specific chromosomes and hormones cooperate at different levels in modulating the multiple steps of the process. Sexual dimorphism also appears to regulate angiogenesis in *in vivo* models. For instance, gender does not affect angiogenesis and tissue reperfusion in a rabbit ischemia model, but estrogen deficiency negatively regulates it (Kyriakides et al., 2003). In a rat model of ischemia/reperfusion injury, the number of chimeric vessels and the total capillary density were higher with transplanted female than male endothelial progenitor cells (Fadini et al., 2008). Female gender or postmenopausal status is a negative predictor of collateral vessel formation in patients with severe coronary artery stenosis (Yetkin et al., 2015). Sex differences have also been recently demonstrated in adipose tissue angiogenesis, with important implications for the development of obesity-related cardiometabolic disease: an increased angiogenic response to high-fat diet in female mice may explain their increased resistance to diet-induced obesity and preserved metabolic homeostasis compared to males (Rudnicki et al., 2018). Overall, evidence of genomic contribution to sex differences in sexually immature animals is lacking. Thus, understanding the pathophysiological relevance of sexually dimorphic angiogenic responses and the interplay with female hormones in different settings deserves additional studies.

To further investigate whether angiogenesis-related sexually dimorphic traits occur through sex hormone-dependent or independent pathways, we focused on the regulation of key signaling molecules involved in the angiogenic process. Estrogenic agents upregulate the functional activity of PFKFB3 (Trenti et al., 2017a), a master regulator of both endothelial cell glucose metabolism and angiogenesis (Schoors et al., 2014; Xu et al., 2014; Peng et al., 2018). This may be in line with established gender differences in glucose homeostasis (Mauvais-Jarvis, 2018). Of note, PFKFB3 is a target of estrogen action in breast cancer cells (Imbert-Fernandez et al., 2014), which have high glycolytic rates similar to endothelial cells. In line with the knowledge that sex differences are mainly due to differential expression of genes present in both sexes (Gershoni and Pietrokovski, 2017), we evaluated PFKFB3 expression in HUVECs from male and female newborns and its role in FBS-induced migration. While PFKFB3 inhibition impaired migration in both mHUVEC and fHUVEC, no sex differences were observed in PFKFB3 baseline levels. In contrast, E2 treatment increased PFKFB3 with higher potency in fHUVECs than in mHUVECs (Figure 6B). Whether gender differences exist in the glycolytic metabolism of endothelial cells remains to be determined.

The functional link between FAK-related pathways and endothelial cell processes relevant to angiogenesis such as motility and proliferation is well established (Sulzmaier et al., 2014). Previous findings from our group showed a central role for FAK phosphorylation at Y576/577 (Trenti et al., 2017b; Trenti et al., 2018) in HUVEC migration and angiogenic potential. Notably, pharmacological inhibition of PFKFB3 reduced FAK Y576/577 phosphorylation and total FAK levels (Supplementary Figure S3), and impaired HUVEC migration, suggesting that the two enzymes are functionally linked and the functional activation of FAK is fostered by glycolysis. The precise mechanism underlying the above link remains to be determined. We herein report for the first time higher levels of FAK phosphorylation at Y576/577 in fHUVEC with respect to mHUVECs. This effect was unrelated to total FAK levels, which were even higher in mHUVECs, and was likely specific for FAK phosphorylation on Y576/577 by Src, which is required for maximal FAK activation (Zhao and Guan, 2011). While the mutual phosphorylation of FAK and Src family tyrosine kinases is required for cell migration (Hanks and Polte, 1997), we did not observe gender-related differences in Src phosphorylation. In addition, while the PI3K/Akt pathway is involved in FBS-induced migration, no gender differences were found in Akt and p-Akt levels (Figure 9B,C). Overall, gender differences in HUVEC migration are mainly related to differences in FAK activation, and such gender differences are abolished by LY294002 (PI3K inhibitor) and 3PO (PFKFB3 inhibitor). The scope for full mechanistic evaluation of these findings, however, goes beyond this current study. Whether other proteins that influence FAK activity and cytoskeleton remodeling, such as the Rho family of GTPases, are differentially expressed in fHUVEC *vs*. mHUVECs remains to be determined. While total FAK was more abundant and phospho-FAK less abundant in mHUVEC vs. fHUVEC, both FAK and phospho-FAK are more expressed in male vs. female vascular smooth muscle cells (Straface et al., 2009), in line with a key role of sex chromosomes in cytoskeleton rearrangement. In addition, consistent with the formation of multiprotein complexes of membrane ERs with Src and FAK, E2 treatment has been reported to induce FAK autophosphorylation at Y397 in HUVECs (Sanchez et al., 2011), although the donors’ sex was not indicated in that study. By contrast, E2 treatment did not change FAK levels in cancer stem cells (Zamani et al., 2020). This implies that, when looking at mechanisms and target proteins involved in functional responses, not only cell origin but also sexual dimorphism needs to be considered.

Identification of sexual dimorphism may be relevant for the outcome of pharmacological treatments targeting endothelial function. For instance, therapeutic agents targeting angiogenic factors such as bevacizumab, a monoclonal antibody that blocks VEGF, display sex differences in outcomes including longer overall survival in female with respect to male patients or sex- and dose-dependent progression-free survival (Brahmer et al., 2011; Reck et al., 2009). The reasons for these differential effects are unclear. Starting from our recent findings that the cardiac glycoside digitoxin at therapeutic concentrations impairs HUVEC migration, capillary tube-like formation and *in vivo* angiogenesis by inhibiting growth factor-induced FAK Y576/577 phosphorylation (Trenti et al., 2017b), we assessed whether such pharmacological responses were affected by HUVEC donors’ sex. In a proof-of-concept experiment, we showed that digitoxin treatment inhibited HUVEC migration in a sex-oriented fashion, with the gender gap being abolished at concentrations within the therapeutic range (Figure 10). The observation that digitoxin treatment does not prevents FAK autophosphorylation at Y397 (Trenti et al., 2017b) rules out overall blockade of cell migration in a FAK-dependent manner. Thus, the above-mentioned sex-specific FAK activation pattern not only contributes to the sexually dimorphic HUVEC angiogenic potential but may also affect the response to agents showing antiangiogenic potential such as digitoxin. These findings highlight the need for sex-oriented pharmacological targeting of endothelial function. Furthermore, since FAK promotes tumor progression through effects on multiple cells of the tumor microenvironment (Sulzmaier et al., 2014; Trenti et al., 2018), interfering with the FAK signaling pathway may be a promising strategy to target invasive cancer types that are prevalent in women (Sood et al., 2004; Lark et al., 2005).

This study has some limitations. First, experiments were performed solely *in vitro*. In addition, due to the heterogeneous pattern of phenotype and ER expression according to vessel type and location, results may not be generalizable to other endothelial cell models. Because of previously published findings from our group and others, responses to estrogenic agents were tested for selected study endpoints only. Despite these limitations, the present findings indicate that the hormonal microenvironment (i.e. estrogen) and donor's sex modulate HUVEC functional phenotypes and signaling molecules involved in angiogenesis by complementary mechanisms that warrant further investigation, and support mechanism-based approaches to gender-specific pharmacological targeting of endothelial function.

## Data Availability Statement

The raw data supporting the conclusions of this article will be made available by the authors, without undue reservation, to any qualified researcher.

## Ethics Statement

The studies involving human participants were reviewed and approved by Ethics Committee, Padua University Hospital. The patients/participants provided their written informed consent to participate in this study.

## Author Contributions

All authors contributed to the study conception and design. Material preparation, data collection and analysis were performed by AT, CaB, CBa, CS, LT, and CBo. The first draft of the manuscript was written by CBo and AC, and all authors commented on previous versions of the manuscript. All authors read and approved the final manuscript.

## Funding

This study was funded by University of Padova PRID Grant 2018 (PI Chiara Bolego). Annalisa Trenti has been recipient of an award from the *Centro Studi Nazionale su Salute e Medicina di Genere* (Padova, Italy).

## Conflict of Interest

The authors declare that the research was conducted in the absence of any commercial or financial relationships that could be construed as a potential conflict of interest.
