# Supplementary material for: Sex Differences in the Pro-Angiogenic Response of Human Endothelial Cells: Focus on PFKFB3 and FAK Activation
Source: Front Pharmacol. 2020 Dec 17;11:587221. doi: 10.3389/fphar.2020.587221 (PMC7773665; doi:10.3389/fphar.2020.587221)
Supplement: Supplementary file 1 [file datasheet1.docx]

Supplementary Material

**Drugs and chemicals**

3PO (3-(3-pyridinyl)-1-(4-pyridinyl)-2- propen-1-one, cat. No. 525330) was purchased from Merck Millipore, Darmstadt, Germany.

**Methods**

**Collective migration assay**

HUVECs (10^5^ cells) were seeded in 24-well plates in complete culture medium. Once reached confluence, the media was replaced with fresh, one scratch was made and cells were incubated in fresh medium containing the tested compounds for additional 6 hours. At the end of the experiment, three images of each well were captured at 4× with a bright field inverted microscope (Nikon Eclipse Ti) equipped with a digital camera, immediately after the scratch was made (time 0) and after 6 hours of incubation. The wound area of each image was measured using ImageJ version 1.47 software (National Institutes of Health, Bethesda, MD), and the average wound area of three images was determined for each sample. Quantitative analysis of cell migration was performed as the percentage of area change using the following formula: % change = [(average space at t0 − average space at t6) ÷ average space at t0] × 100. Values are expressed as % change from cells at T0.

**Western blot and chemotaxis assay**

These assays were carried out as described in the main text.

**Results**

**Supplemental Figure 1.** Effect of donor’s sex on HUVEC collective migration.

**
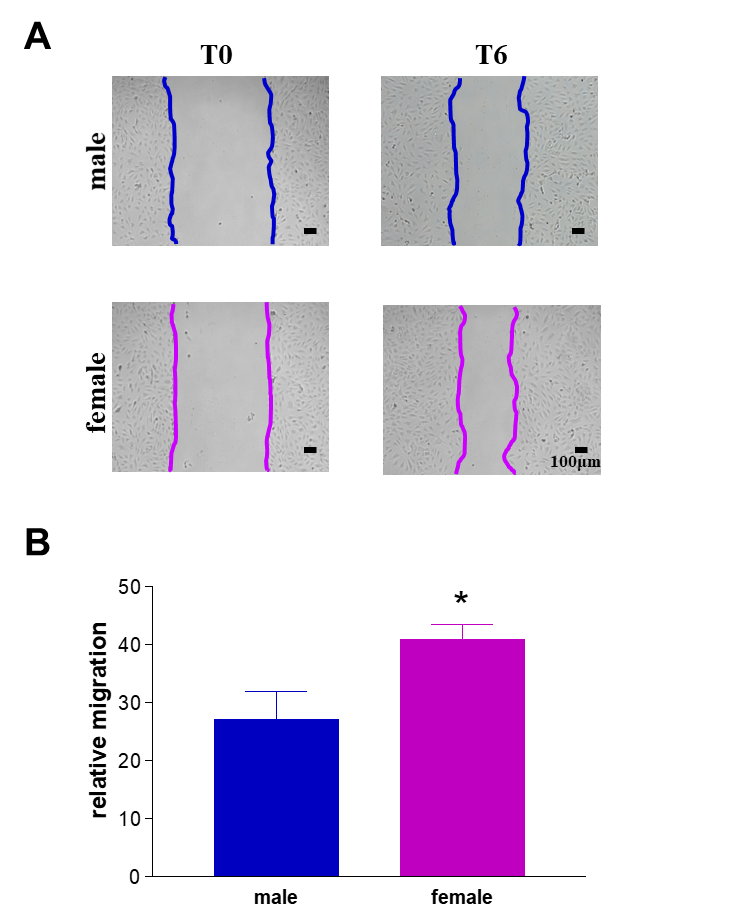
**

HUVECs from male and female donors (10^5^ cells/well) were seeded in 24-well plates and the assay was performed in confluent cells. Thereafter, monolayers were wounded (t0), and treated as above for 6 hours (t6). Cell migration was calculated as described in Methods. **A**. Representative image of a wound healing experiment (4x magnification, scale bar: 100 µm). **B**. Quantitative analysis of wound healing experiments. Each independent experiment (*n*=3) was performed using cells from 1 male and 1 female donor. Data are expressed as mean ± SEM. *t*-test, * *P* < 0.05.

**Supplemental Figure 2:** Effect of the PFKFB3 inhibitor 3PO on male and female HUVEC migration in a chemotaxis assay using 15% FBS as chemoattractant agent.


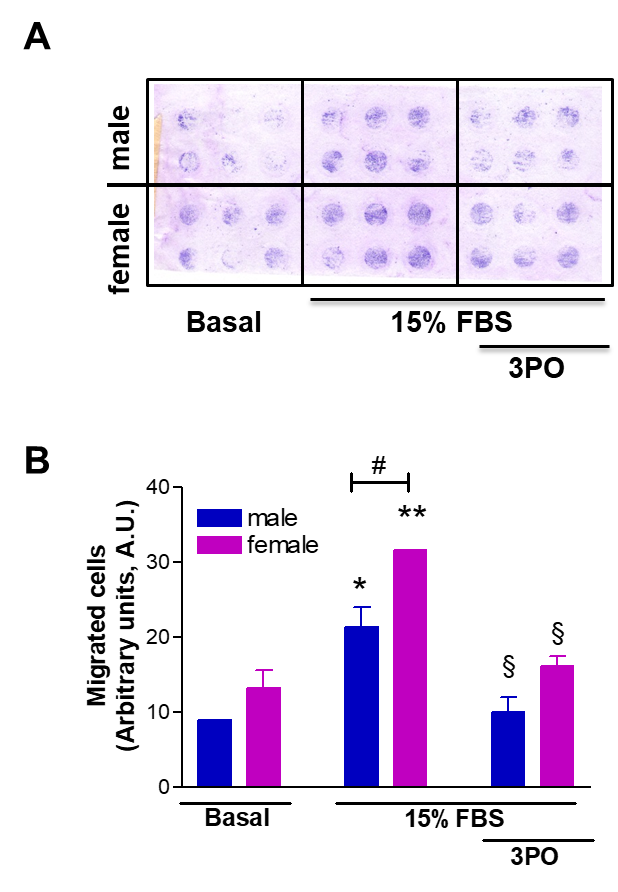


**A**. Representative image of FBS-induced HUVEC migration in response to the PFKFB3 inhibitor 3PO (40 µM) in a modified 48-well Boyden chamber after 6 hours’ incubation at 37°C. **B**. Cell migration is shown as optical density values (A.U., arbitrary units). Each independent experiment (*n*=3) was performed in sextuplicate using cells from 1 male and 1 female donor. Data are expressed as mean ± SEM *t*-test; * *P* < 0.05, ** *P* < 0.01 (*vs* basal); ^#^ *P* < 0.05; § *P* < 0.05 (*vs* 15% FBS alone).

**Supplemental Figure 3:** Effect of the PFKFB3 inhibitor 3PO on FAK activation


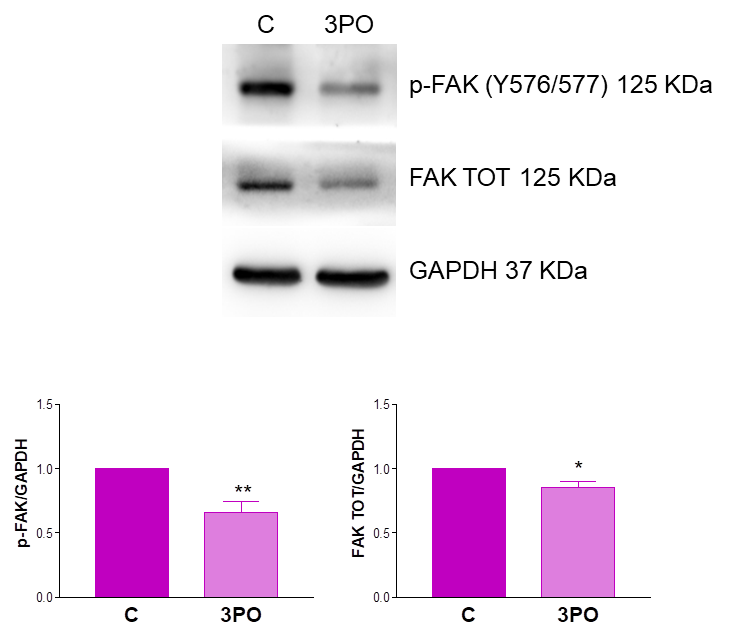


HUVECs from female donors were seeded in 35-mm dishes in M199 complete medium and, after reaching confluence, treated with 3PO (40 µM) or vehicle for 6 h. *Upper panels:* Representative Western blots showing the expression of p‐FAK (Y576/577) and total FAK; GAPDH was used as loading control. *Lower panels*: Densitometric analysis of bands normalized to GAPDH levels. The C value was set as 1. Data are shown as mean ± SEM of 4 independent experiments. *t*-test, * *P* < 0.05; ** *P* < 0.01.
